# Analysis of health system characteristics needed before performance assessment

**DOI:** 10.2471/BLT.24.291760

**Published:** 2024-06-04

**Authors:** Ruth Waitzberg, Isabel Deborah Pfundstein, Anna Maresso, Bernd Rechel, Ewout van Ginneken, Wilm Quentin

**Affiliations:** a Department of Health Care Management, Berlin University of Technology, Berlin, Germany.; b University Geriatric Medicine FELIX PLATTER, Basel, Switzerland.; c European Observatory on Health Systems and Policies, Berlin University of Technology, Berlin, Germany.; d European Observatory on Health Systems and Policies, London School of Hygiene & Tropical Medicine, London, England.; e German West-African Centre for Global Health and Pandemic Prevention, Chair of Planetary & Public Health, University of Bayreuth, Universitätsstrasse 30, 95447 Bayreuth, Germany.

To assess the performance of a health system, understanding its structures and functions is necessary. This understanding requires an in-depth description and analysis of the health system, which can be facilitated using standardized assessment templates. While many national and international actors work on health systems strengthening, they often struggle to find reliable systematic information on the design and functioning of a health system. At the same time, national policy-makers might seek to learn from experiences from other systems and contexts, but do not always find comparable information on other countries’ health systems. Using a standardized guide or template when describing and assessing how a health system functions can support cross-country comparisons because the structured nature of a template simplifies the extraction of comparable information.^1^^,^^2^ Several international agencies, including the World Health Organization (WHO), the Organisation for Economic Co-operation and Development (OECD), the United States Agency for International Development (USAID), the European Union (EU) and the Commonwealth Fund have developed such templates. We have reviewed 12 of these templates (Waitzberg R, Berlin University of Technology, unpublished material, 2024) and believe that there is much scope for improvement and harmonization (Box 1). Templates were defined as having an overall framework, a list of indicators or topics and instructions for users, while covering the entire health system and the design of the health system, as well as including an assessment of health system performance.

Box 1 **Templates found in an 2023 scoping review**

1. A common evaluation framework for the African Health Initiative (2013).^3^

2. Commonwealth Fund health profiles (2020).^4^

3. African Health Observatory Platform on Health Systems and Policies Country Health Systems and Service Profile: An overview (2020).^5^

4. European Observatory on Health Systems and Policies - Health Systems in Transition template for authors (2019).^6^

5. Health Systems in Action insights. European Observatory on Health Systems and Policies (2021).^7^

6. Monitoring Framework for Universal Health in the Americas, Pan American Health Organization (2021).^8^

7. OECD health systems characteristics survey, Latin American Countries (2018).^9^^,^^10^

8. Pan American Health Organization/WHO/USAID Health Systems Country Profiles 1999–2009.^11^

9. State of Health in the EU – country health profiles (2019).^12^^,^^13^

10. USAID UHC Monitoring Framework with Ethiopia country report as a case study (2017).^14^

11. USAID’s health system assessment approach: a how-to manual, version 3.0 (2017).^15^

12. Monitoring the building blocks of health systems: a handbook of indicators and their measurement strategies (2010).^16^

EU: European Union; OECD: Organisation for Economic Co-operation and Development; WHO: World Health Organization; UHC: Universal Health Coverage; USAID: United States Agency for International Development. Source: Waitzberg R, Berlin University of Technology, unpublished material, 2024.

## Information gaps

While the structures and processes of health systems differ across countries, all perform the same essential functions of governance; health financing; ensuring the availability of medical products, vaccines and technologies; generating relevant health information; creating and sustaining a health workforce; and providing health services.^17^ Policy-makers, advisors and researchers can learn much from deep, systematic descriptions of these functions and use them to interpret the results of health system performance assessments. Templates that guide authors on how to fully describe health systems should cover all these essential functions.

While existing templates cover some functions, they do not cover all of them. For example, health financing is always covered, and service delivery, health workforce and governance are frequently covered, albeit sometimes under different labels. However, health information systems and medical products are often missing. Similarly, the way health system performance is addressed in existing templates is incomplete as it is frequently assessed regarding access and coverage, quality and safety and financial protection, but less often with regard to responsiveness and efficiency.

Yet, these topics are important and should be better explored across all countries and thus covered in all templates. For example, the lack of attention to health information systems is surprising, given their central role in generating data that can be used to describe and steer health systems.^18^^,^^19^ While measuring the performance of health systems in achieving their goals is challenging, it should form an integral part of descriptions and analyses of health systems. Health systems aim to improve efficiency and responsiveness, and these objectives therefore deserve more attention in health system templates. International organizations could join forces and create a unified template where they agree on core indicators and core topics to be covered, as well as the main methods of data collection. Such a publication could, for example, build on the collaboration between WHO, OECD and the European Observatory on Health Systems and Policies’ *Health system performance assessment: a renewed global framework for policy-makin*g.^20^

## Health systems indicators

Various agencies have developed core health indicators to describe the functions and assess the performance of health systems. For example, the WHO Regional Office for the Eastern Mediterranean has 84 core health indicators;^21^ the EU has more than 60 core health indicators;^22^ WHO has proposed 17 core indicators for monitoring building blocks;^16^ and the global Health Systems Performance Assessment dashboard has 24 key indicators for two functions and two goals of the health system.^23^ While all templates suggest that users describe systems with various indicators (on average 53 indicators per template), only 11 indicators are used in at least half of the templates and can be considered frequently used (Pfundstein ID, University Geriatric Medicine FELIX PLATTER, unpublished material, 2024). A more standardized list of core indicators for templates would be useful.

We searched for the availability of the 11 indicators in 125 countries in the WHO regions of the Americas, Eastern Mediterranean and Europe in 2000–2023, resulting in a total of 1375 indicators. About 80% (1099) of frequently used indicators in templates were available. However, many important indicators are less available.^11^ Indicators on service delivery are missing in many templates, which can be explained by the lack of these data in international databases, such as in the WHO African Region.^24^ Therefore, building an evidence base on the functioning and performance of health systems worldwide requires greater efforts to promote data availability for a standardized set of indicators.

## Accounting for contextual differences

Different world regions struggle with different problems, both external and internal to the health system, including different economic, geographic and political contexts, population structures and burdens of disease. Some health systems face difficulties related to crowded cities, while others struggle to reach remote areas. Different health systems cope with varying degrees of fragmentation in the organization or delivery of care, different roles of private provision or funding, different patient pathways, and different responsibilities and remits of health workers.

Health system analyses must consider these contextual differences, for example by including in templates optional topics or indicators that may be more relevant to some countries than others. Furthermore, templates may recommend different sources of data for the same indicators. For example, high-income countries tend to have more data on routine health service utilization and data from civil registration systems, while low- and middle-income countries tend to have data for similar indicators from population or facility surveys.

## The need for qualitative data 

A comprehensive health system analysis and comparison requires a combination of quantitative indicators complemented with systematic qualitative information that captures non-measurable characteristics. For example, qualitative information adds value on the degree of decentralization of service delivery, the governance of providers, the payment methods used and the skills of health workers. Qualitative information is particularly suitable to capture processes, changes and outcomes, while an (over-) reliance on quantitative indicators may result in comparisons limited to quantifiable parameters. Recent work focusing on health system performance suggests combining quantitative with qualitative data to enrich assessments.^1^^,^^25^

## Conclusion

Achieving comparable, standardized information on health system structures, processes and outcomes at the global level requires templates with greater standardization and better harmonization of indicators, and a greater availability of health system data. Such templates would allow the systematic analysis of health system functions, and make the results of health system performance assessments more useful to policy-makers and researchers. The resulting in-depth understanding of health systems is crucial for efforts to strengthen health systems because in complex adaptive systems, changes to one function will have implications for all other functions.
